# Antifungal Activity, Toxicity and Chemical Composition of the Essential Oil of *Coriandrum sativum* L. Fruits

**DOI:** 10.3390/molecules17078439

**Published:** 2012-07-11

**Authors:** Bruna V. Soares, Selene M. Morais, Raquel Oliveira dos Santos Fontenelle, Vanessa A. Queiroz, Nadja S. Vila-Nova, Christiana M. C. Pereira, Edy S. Brito, Manoel A. S. Neto, Erika H. S. Brito, Carolina S. P. Cavalcante, Débora S. C. M. Castelo-Branco, Marcos F. G. Rocha

**Affiliations:** 1 Postgraduate Program in Veterinary Sciences, State University of Ceará, 60740-000, Fortaleza, CE, Brazil; E-Mails: drbrunasoares@yahoo.com.br (B.V.S.); nadja.vilanova@hotmail.com (N.S.V.-N.); 2 Department of Chemistry, State University of Ceará, 60740-000, Fortaleza, CE, Brazil; E-Mails: selene@uece.br (S.M.M.); vanessa_videl@hotmail.com (V.A.Q.); chirstianacoelho@hotmail.com (C.M.C.P.); 3 Centre of the Agricultural Sciences and Biological, Acaraú Valley State University, 62040-370, Sobral, CE, Brazil; 4 Embrapa Tropical Agroindustry Center, Fortaleza, 89700-000, Ceará, Brazil; E-Mails: Edy@cnpat.embrapa.br (E.S.B.); Manoel@cnpat.embrapa.br (M.A.S.N.); 5 Department of the Veterinary, Faculty of Veterinary Medicine, Superior Institute of Applied Theology, 62050-100, Sobral, CE, Brazil; E-Mails: sallesbrito@yahoo.com.br (E.H.S.B.); carolinaspc@yahoo.com.br (C.S.P.C.); 6 Department of Pathology and Legal Medicine, School of Medicine, Specialized Medical Mycology Center, Federal University of Ceará, Fortaleza, 60441-750, Ceará, Brazil; E-Mails: deb_castelobranco@yahoo.com (D.S.C.M.C.-B.); mfgrocha@gmail.com (M.F.G.R.)

**Keywords:** *Coriandrum sativum*, essential oil, *Microsporum canis*, *Candida* spp., *Artemia salina*, linalool

## Abstract

The aims of this study were to test the antifungal activity, toxicity and chemical composition of essential oil from *C. sativum* L. fruits. The essential oil, obtained by hydro-distillation, was analyzed by gas chromatography/mass spectroscopy. Linalool was the main constituent (58.22%). The oil was considered bioactive, showing an LC_50_ value of 23 µg/mL in the *Artemia salina* lethality test. The antifungal activity was evaluated against *Microsporum canis* and *Candida* spp. by the agar-well diffusion method and the minimum inhibitory concentration (MIC) and the minimum fungicidal concentration (MFC) were established by the broth microdilution method. The essential oil induced growth inhibition zones of 28 ± 5.42 and 9.25 ± 0.5 for *M. canis* and *Candida* spp. respectively. The MICs and MFCs for *M. canis* strains ranged from 78 to 620 and 150 to 1,250 µg/mL, and the MICs and MFCs for *Candida* spp strains ranged from 310 to 620 and 620 to 1,250 µg/mL, respectively. *C. sativum* essential oil is active *in vitro* against *M. canis* and *Candida* spp. demonstrating good antifungal activity.

## 1. Introduction

Dermatophytosis is one of the most frequent skin diseases of pets and livestock. Contagion among animal communities, high treatment cost, difficulties of control measures and public health consequences of animal ringworm are all factors urging the study of these fungi [1]. Fungal disease agents are widespread and can be isolated from a wide range of sick animals or asymptomatic carriers, which can represent important reservoirs for people in close contact with them. This situation should be considered as an important risk factor for those with impaired immune systems and anyone working with or handling animals. The incidence of dermatophytosis caused by *Microsporum canis* is increasing in human patients in many places around the World, including several Brazilian cities, and it is often the predominant fungus seen in dermatological clinics [2].

Yeasts of the *Candida* genera can be found as commensal microorganisms in animals and are considered one of the most important species in veterinary medicine. Strains of *Candida* spp. isolated from dogs showed high resistance to azole antifungal agents [3]. Although effective antimicrobials have been developed over the years, there has been increased development of antimicrobial drug resistance to currently available antimicrobials [4]. Many essential oils and plant extracts used in therapy have advantages over antibiotics, although the latter are more effective [5].

The essential oil from leaves of *C. sativum* showed antimicrobial activity against both Gram positive and Gram negative bacteria. This plant is known not to be toxic because it has been consumed for centuries without showing any signs of toxicity [6].

Due to known antibacterial activity of the leaf essential oil of *C. sativum*, the aim of this study was to evaluate the antifungal activity of the essential oil from fruits by the agar diffusion and microdilution methods, and to determine its main chemical constituents. Also, to evaluate the potential use of *C. sativum* essential oil as a phytotherapic product, the toxicity was investigated using the *Artemia salina* lethality test.

## 2. Results and Discussion

The chemical analysis of the *C. sativum* is shown in Table 1. The main constituents of the essential oil of *C. sativum* were linalool (58.65%), geraniol (17.87%) and neryl acetate (12.22%). The brine shrimp lethality test of the essential oil showed an LC_50_ of 23 µg·mL^−1^, being considered bioactive, and the estimated LD_50_ obtained by linear regression was 2,139.98 mg/kg.

The essential oil from *C. sativum* fruits was effective against all tested fungal strains in the agar-well diffusion susceptibility tests (Table 2). The oil induced a significant growth inhibition zone (28 ± 5.42 mm) at a concentration of 10,000 µg/mL against *M. canis* strains (n = 4). For *Candida* strains (n = 4), the growth inhibition zone induced by the oil was 9.25 ± 0.5 mm, at a concentration of 10,000 µg/mL. The positive control, griseofulvine, induced a significant growth inhibition zone (55.25 ± 3.69 mm) against *M. canis* and amphotericin B induced a significant growth inhibition zone (10.25 ± 1.26 mm) against *Candida* spp.

The broth microdilution method showed that the MICs for *M. canis* strains (n = 5) ranged from 78 to 620 µg/mL and the MFCs ranged from 150 to 1,250 µg/mL. The MICs for *Candida* spp. strains (n = 5) ranged from 310 to 620 µg/mL and the MFCs varied from 620 to 1,250 µg/mL (Table 3).

Many essential oils have been advocated for use in complementary medicine for bacterial and fungal infections [7]. Previous studies have investigated the activity of essential oils against dermatophytes and yeasts [5,6,7]. In our ongoing search for new antimicrobial agents, the essential oil of *C. sativum* was tested against animal fungal strains and produced good results. 

The antifungal activity of the essential oil of *C. sativum* fruits may be attributed to its main constituents, linalool (58.22%) and geraniol (17.87%). Previous studies of the chemical composition of *C. sativum* fruit essential oil carried out by Pino *et al.* [8] and Burt [9] reported linalool concentrations of 54.57% and 70%, respectively. Antimicrobial activity of linalool against several bacteria and fungi has also been reported [10,11,12]. Dorman and Deans [13] tested the antimicrobial activity of some essential oils and their main chemical constituents. Among them, linalool and geraniol were individually effective against 23 different bacterial strains.

Linalool and linalyl acetate are monoterpenoid compounds that are common in many essential oils of several aromatic species. A number of linalool and linalyl acetate producing species are used in traditional medicinal systems to relieve symptoms and cure a variety of ailments, both acute and chronic [14].

The essential oil from *C. sativum* leaves showed antimicrobial activity against Gram positive (*Staphylococcus aureus*, *Bacillus* spp.) and Gram negative bacteria (*Escherichia coli*, *Salmonella typhi*, *Klebsiella pneumonia*, *Proteus mirabilis*) and a pathogenic fungus (*Candida albicans*). The main constituents revealed by chemical analysis were 2(*E*)-decenal (15.9%), decanal (14.3%), 2(*E*)-decen-1-ol (14.2%) and *n*-decanol (13.6%) [6]. According to these authors, the MIC against *C. albicans* was 163 mg/mL and the oil from fruits in that study was 434 µg·mL^−1^. The essential oil of *C. sativum* fruits exhibits higher antifungal activity against *Candida* spp. Begnami *et al*. [15] reported the antifungal activity of this plant against different *Candida* species. They suggested that the essential oil of this plant could be used as potential antimicrobial agents to treat or prevent *Candida* yeast infections. This is corroborated by our findings. Silva *et al*. [16] found an increasing incidence of drug-resistant pathogens and toxicity of existing antifungal compounds while studying the synergism of this essential oil with amphotericin B against *Candida* species. Their results suggest that the essential oil of *C. sativum* could be useful in designing new formulations for candidosis treatment.

In veterinary practice, dermatophytoses are among the most common infectious skin diseases in mammals worldwide. They are frequently observed in domestic animals, but also in captive and wild fauna [1]. Based on the results of this study, the essential oil obtained from fruits of *C. sativum* could be an alternative natural source to treat animal dermatophytoses.

In the evaluation of plant extract toxicity by the brine shrimp bioassay, an LC_50_ value lower than 1,000 µg/mL is considered bioactive [17]. In this study, the essential oil from *C. sativum* fruits showed an LC_50_ value of 23 µg/mL. This result corroborates the antifungal properties of the oil. Parra *et al.* [18] assessed the effect of acute treatment of *A. salina* larvae and mice with several extracts drawn from autochthonous plants in Cuba. The aim of their study was to develop a low-cost method applicable to countries where the use of medicines obtained from vegetable species is common and is an affordable way to fight diseases. They calculated LC_50_ values for *A. salina* larvae and LD_50_ values for mice and established significant correlations between both parameters, suggesting the use of *A. salina* larvae as a suitable, accurate and inexpensive alternative to pre-screening chemical toxicity with mammals [19]. The estimated LD_50_ in mice for *C. sativum* essential oil was 2,139.98 mg/kg and this value indicates a low toxicity in accordance with Hedge and Sterner [20].

## 3. Experimental 

### 3.1. Plant Material and Extraction of Essential Oils

The fruits used for extraction of the essential oil were harvested from plants cultivated in the Medicinal Plants Orchard of State University of Ceará, from commercial seeds produced by ISLA Sementes Ltda. (Porto Alegre, RS, Brazil). The *C. sativum* essential oil was extracted by the hydro-distillation method in a modified Clevenger apparatus, as described by Craveiro *et al.* [21].

### 3.2. Gas-Chromatography/Mass Spectral (GC-MS) Analysis

The chemical analysis of the essential oil constituents was performed on a Shimadzu QP-2010 instrument employing the following conditions: column: DB-5ms (Agilent, part No. 122-5532) coated fused silica capillary column (30 m × 0.25 mm × 0.25 µm); carrier gas: He (1 mL/min, in constant linear velocity mode); injector temperature was 250 °C, in split mode (1:100), and the detector temperature was 250 °C. The column temperature programming was 35 to 180 °C at 4 °C/min then 180 to 280 °C at 17 °C/min, and at 280 °C for 10 min; mass spectra: electron impact 70 eV. The injected sample volume was 1 µL. Compounds were identified by their GC retention times relative to know compounds and by comparison of their mass spectra with those present in the computer data bank (National Institute for Standard Technology–NIST–147, 198 compounds) and published spectra [22,23].

### 3.3. Brine Shrimp Lethality Bioassay

The essential oil of *C. sativum* was assayed using a modified test of lethality to *A. salina* [17]. The eggs of *A. salina* were incubated in a hatching chamber with seawater and kept at room temperature (average 27 °C) under artificial light around the clock. Larvae after 48 h were extracted and counted using a Pasteur pipette. A standard solution of 1,000 µg/mL was prepared with 100 mg of essential oil diluted in 1.0 mL of DMSO, and the volume was completed with seawater in a 100 mL volumetric flask. Concentrations of 900, 100, 10 and 1 µg/mL were prepared using standard solution. For each concentration, 10 brine shrimp larvae were used, placed in flasks that were filled with seawater to a total volume of 5 mL. Intermediate concentrations were made to calculate the LC_50_. For the control group, a solution was prepared with 100 µL of DMSO and 4.9 mL of seawater. After 24 h, the dead larvae were counted and the LC_50_ value was estimated using the Origin 7.0 statistical program.

### 3.4. LD_50_ Estimate Calculation for *C. sativum* Essential Oil

The LD_50_ value was based on the comparative study of the assay of *A. salina* and the lethal dose (LD_50_) value in mice, to determine acute oral toxicity of plant extracts [18]. The published LC_50_ and LD_50_ values of the extracts were correlated using the Origin 7.0 statistical program to obtain the linear regression equation Y = 169.57 + 85.67X (R = 0.86), where Y is the LD_50_ value, X is the LC_50_ value and R is the correlation coefficient. The LD_50_ figure was expressed in mg/Kg.

### 3.5. Fungal Strains

A total of five strains of *M. canis* and five strains of *Candida* spp. were included in this study. Both *M. canis* and *Candida* spp. strains were isolated from symptomatic dogs and cats. The strains were stored in the fungal collection of the Specialized Medical Mycology Center-CEMM (Federal University of Ceará, Brazil), where they were maintained in saline (0.9% NaCl), at 28 °C. At the time of the analysis, an aliquot of each suspension was taken and inoculated into potato dextrose agar (Difco, Detroit, MI, USA), and then incubated at 28 °C for 2–10 days.

### 3.6. Inoculum Preparation for Antifungal Susceptibility Tests

For the agar-well difusion method, based on Fontenelle *et al.* [5], stock inocula were prepared on day 2 and day 10 for *Candida* spp. and *M. canis*, respectively, grown on potato dextrose agar (Difco) at 28 °C. Potato dextrose agar was added to the agar slant and the cultures were gently swabbed to dislodge the conidia. The suspensions with blastoconidia of *Candida* spp. or suspension of hyphal fragments of *M. canis* were transferred to a sterile tube and adjusted by turbidimetry to obtain inocula of approximately 10^6^ cfu/mL blastoconidia of *Candida* spp. and 10^5^ cfu/mL hyphal fragments or conidia of *M. canis.* The optical densities of the suspensions were spectrophotometrically determined at 530 nm and then adjusted to 95% transmittance. 

For the broth microdilution method, standardized inocula (2.5–5 × 10^3^ cfu/mL for *Candida* spp. and 5 × 10^4^ cfu/mL for *M. canis*) were also prepared by turbidimetry. Stock inocula were prepared on day 2 and day 10 for *Candida* spp. and *M. canis* cultures, respectively, grown on potato dextrose agar at 28 °C. Sterile normal saline solution (0.9%; 3 mL) was added to the agar slant and the cultures were gently swabbed to dislodge the conidia from the hyphal mat for the *M. canis* [24] and the blastoconidia from *Candida* spp. [3]. The suspensions of conidia with hyphal fragments of *M. canis* and blastoconidia suspension of *Candida* spp. were transferred to a sterile tubes, and the volume of both suspensions adjusted to 4 mL with sterile saline solution. The resulting suspension were allowed to settle for 5 min at 28 °C, and their density was read at 530 nm and the adjusted to 95% transmittance. The suspensions were diluted to 1:2,000 for *Candida* spp. and 1:500 for *M. canis*, both with RPMI 1640 medium (Roswell Park Memorial Institute-1640) with L-glutamine, without sodium bicarbonate (Sigma Chemical Co., St. Louis, MO, USA), buffered to pH 7.0 with 0.165 M morpholinopropanesulfonic acid (MOPS) (Sigma Chemical Co.), to obtain the inoculum size of approximately 2.5–5 × 10^3^ cfu/mL for *Candida* spp. and 5 × 10^4^ cfu/mL for *M. canis*.

### 3.7. Agar-Well Diffusion Susceptibility Test

The antifungal activity of essential oil from *C. sativum* was evaluated against *Candida* spp. (n = 4) and *M. canis* (n = 4), by the agar-well diffusion method according to Fontenelle *et al.* [5]. Petri dishes with 15 cm diameter were prepared with potato dextrose agar (Difco). The wells (6 mm in diameter) were then cut from the agar and 100 µL of essential oil was delivered into them. The oil was weighed and dissolved in DMSO to obtain the test concentration of 10,000 µg/mL. Stock solutions of griseofulvin (1,000 µg/mL; Sigma Chemical Co.) and amphotericin B (5 µg/mL; Sigma Chemical Co.) were prepared in distilled water and tested as positive controls for *M. canis* and *Candida* spp., respectively. Each fungal suspension was inoculated on to the surface of the agar. After incubation, for 3–5 days for *Candida* spp. and 5–8 days for *M. canis*, at 28 °C, all dishes were examined for zones of growth inhibition and the diameters of these zones were measured in millimeters. Each experiment was repeated at least twice.

### 3.8. Broth Microdilution Method

The minimum inhibitory concentration (MIC) for *Candida* spp. was determined by the broth microdilution method, in accordance with the Clinical and Laboratory Standards Institute-CLSI (formerly NCCLS; M27-A2), [25]. The broth microdilution assay for *M. canis* was performed as described by Brilhante *et al.* [24], based on the M38-A document (CLSI; formerly NCCLS, 2002) [26]. The minimum fungicidal concentration (MFC) for both *Candida* spp. and *M. canis* were determined according Fontenelle *et al.* [5]. In addition, *C. parapsilosis* (ATCC 22019) and *C. albicans* (ATCC 1023) strains were used as quality controls for broth microdilution method.

The essential oil of *C. sativum* was prepared in DMSO. Amphotericin B (AMB) (Sigma Chemical Co.) and griseofulvine (Sigma Chemical Co.) were prepared in distilled water. For the susceptibility analysis, the essential oils were tested in concentrations ranging from 4 to 5,000 µg/mL.

The microdilution assay was performed in 96-well microdilution plates. Growth and sterile control wells were included for each isolate tested. The microplates were incubated at 37 °C and read visually after 2 days for *Candida* spp. and 5 days for *M. canis*. The assays for all essential oils were run in duplicate and repeated at least twice. The MIC was defined as the lowest oil concentration that caused 100% inhibition of visible fungal growth. The results were read visually as recommended by CLSI. The MFC was determined by subculturing 100 µL of solution from wells without turbidity, on potato dextrose, at 28 °C. The MFCs were determined as the lowest concentration resulting in no growth on the subculture after 2 days for *Candida* spp. and 5 days for *M. canis*.

### 3.9. Statistical Analysis

Antifungal activity was expressed as mean ± SD of the diameter of the growth inhibition zones (mm). The antifungal activity of the essential oils was analyzed by linear correlation for individual analysis and the two-tailed Student’s *t*-test at 95% confidence intervals was used to evaluate differences between the essential oil and the controls. For the brine shrimp lethality bioassay, the LC_50_ and the LC_50_ values were estimated using the Origin 7.0 statistical program. 

## 4. Conclusions

Owing to its broad spectrum of antifungal effect, *in vitro*, and low toxicity, the essential oil of *C. sativum* is a promising source in the search for new antifungal drugs. However, it is necessary to evaluate the acute toxicological effects and antifungal efficacy *in vivo* in order to be considered for a safe and effective antifungal agent.

## Figures and Tables

**Table 1:** Chemical composition of the essential oil from *C. sativum*.

K.I. *	Components	Composition (% **)
**1098**	**Linalool**	**58.22**
1144	Camphor	2.15
1168	Borneol	1.19
1203	n-Decanal	2.53
**1247**	**Geraniol**	**17.87**
1258	2E-Decenal	1.32
**1373**	**Neryl acetate**	**12.22**
1405	Dodecanal	2.35
1462	2E-Dodecenal	0.95

* Retention index. The identified constituents are listed in their order of elution from a nonpolar column; ** The % composition is the % peak area of the total essential oil composition.

**Table 2:** Antifungal activity of the essential oil from *C. sativum* against *Microsporum canis* and *Candida* spp. in the agar-well diffusion assay.

Strains	Growth inhibition zones (mm)		
	*C. sativum* essential oil (µg/mL)	Griseofulvine (µg/mL)	Amphotericin B (µg/mL)
	10,000	1,000	5
***M. canis***			
CEMM 01-5-190	30	60	-
CEMM 01-4-104	20	55	-
CEMM 01-3-188	32	55	-
CEMM 01-3-186	30	51	-
(mean ± SD)	(28 ± 5.42)	(55.25 ± 3.69)	-
***Candida* spp.**			
CEMM 01-3-077	9	-	12
CEMM 01-3-069	9	-	10
CEMM 01-2-078	10	-	10
CEMM 01-2-081	9	-	09
(mean ±SD)	(9.25 ± 0.5)	-	(10.25 ± 1.26)

Each experiment was performed in duplicate.

**Table 3:** Minimum inhibitory and fungicidal concentrations of *C. sativum* essential oil against *M. canis* and *Candida* spp.

Strains	*C. sativum* essential oil	
	MIC (µg/mL)	MFC (µg/mL)
***M. canis***		
CEMM 01-5-190	78	150
CEMM 01-4-104	310	620
CEMM 01-3-188	620	1,250
CEMM 01-3-186	620	1,250
CEMM 01-3-165	620	1,250
(Geometric mean)	449.6	904
***Candida* spp.**		
CEMM 01-3-077	620	1,250
CEMM 01-3-069	310	620
CEMM 01-2-078	310	620
CEMM 01-2-081	620	1,250
CEMM 01-3-068	310	620
(Geometric mean)	434	872

MIC: Minimum inhibitory concentration expressed in µg·mL^−1^; MFC: Minimum fungicidal concentration expressed in µg·mL^−1^; CEMM: Specialized Medical Mycology Center. Each experiment was repeated at least twice. Broth microdilution method.
